# Primary Squamous Cell Carcinoma of the Sigmoid Revealed by Acute Intestinal Occlusion in Moroccan Young Male Patient: A Case Report and Literature Review

**DOI:** 10.7759/cureus.34283

**Published:** 2023-01-27

**Authors:** Mohamed Amine Haouane, Jouabri Badr, Mohamed Kaakoua, Mohamed Amine Azami

**Affiliations:** 1 Department of Pathology, Caddi Ayyad University of Marrakech/Ibn Sina Military Hospital, Marrakech, MAR; 2 Department of General Surgery, Caddi Ayyad University of Marrakech/Ibn Sina Military Hospital, Marrakech, MAR; 3 Department of Medical Oncology, Caddi Ayyad University of Marrakech/Ibn Sina Military Hospital, marrakech, MAR

**Keywords:** surgery, intestinal occlusion, squamous cell carcinoma (scc), cancer, colon

## Abstract

Primary colorectal squamous cell carcinoma (SCC) is an extremely rare subtype of colon cancer, with an incidence of less than 1% of colorectal malignancies. We report a case of a 40-year-old male patient admitted to the emergency department with symptoms of acute intestinal obstruction. Histopathological evaluation of colonoscopic biopsies revealed squamous cell carcinoma. A sigmoidectomy was performed.

In order to enrich the medical literature, we add our case to the collection of colorectal squamous cell carcinoma cases by analyzing and summarizing the clinical, pathological, and therapeutic features of this rare entity.

## Introduction

Colorectal cancer (CRC) is one of the most common malignancies worldwide. Adenocarcinoma is the most common histological type and accounts for more than 90% of all colorectal malignancies [1]. However, primary squamous cell carcinoma (SCC) of the colon is an extremely rare malignancy and accounts for 0.02-0.1% of all colorectal cancers [2]. The etiology, clinical manifestations, biological behavior, treatment, and prognosis remain unclear. As a consequence, more case reports are needed to completely comprehend the SCC of the large intestine [3].

Herein, we reported a case of primary SCC of the sigmoid colon revealed by acute intestinal occlusion and a brief review of the literature regarding this extremely rare entity.

## Case presentation

A 40-year-old man with four days of abdominal pain, distension, vomiting, and constipation was admitted to the emergency room. The patient had no previous medical history or a family history of colonic cancers. On examination, his abdomen was soft, minimally tender all over, with slight distension. All blood test results were normal. Abdominal x-ray demonstrated the existence of dilated large bowel with air-fluid levels.

**Figure 1:**
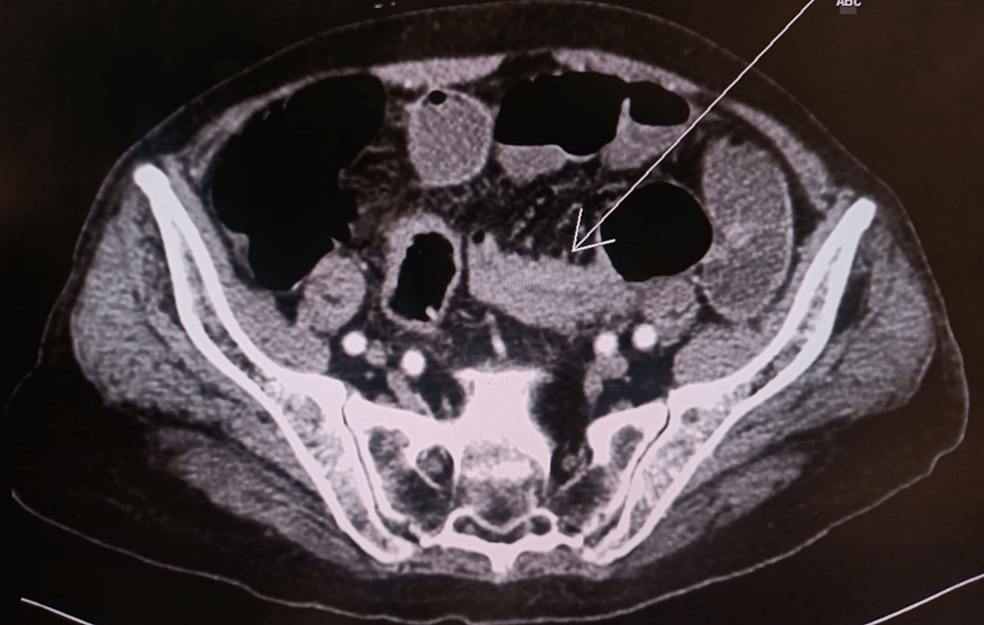
Abdominal CT scan showing a large obstructing solid mass arising from the sigmoid colon (arrow), measuring approximately 6 cm x 5.5 cm.

A computed tomography (CT) of the abdomen was performed and revealed a large circumferential and stenosing mass of the sigmoid measuring approximately 6x5.5 cm (Figure 1). At that time, no other distant metastatic disease was visible in the abdomen or pelvis.

The patient was diagnosed with colonic obstruction caused by a sigmoid tumor. The patient underwent a laparotomy with loop colostomy to relieve his intestinal obstruction. One day after his admission a total colonoscopy was recommended, which discovered the presence of a large obstructing and circumferential mass with infiltrative growth in the sigmoid colon approximately 40 cm from the anus. The anal squamous epithelium was normal without any lesions or ulcers. A biopsy of the tumor was performed with a colonoscopy.

**Figure 2:**
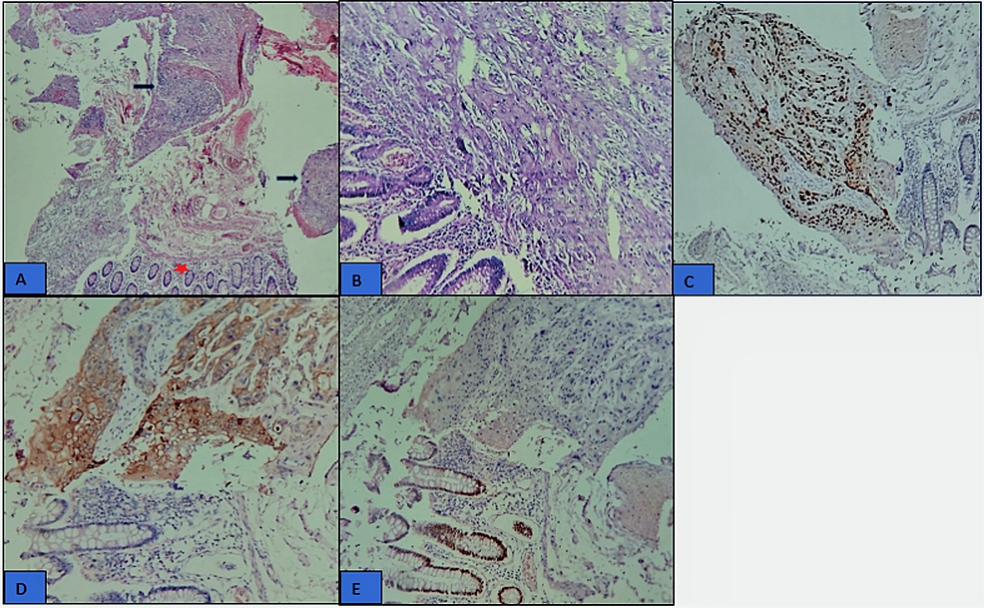
Photomicrograph image showing histological with hematoxylin and eosin stain and immunohistochemical features of squamous cell carcinoma of the sigmoid: (A) (Low magnification) showing normal colonic mucosa (red star) with adjacent moderately differentiated keratinizing squamous cell carcinoma (black arrow). (B) (High magnification) showing large polygonal tumor cells, showing hyperchromatic, anisocaryotic nuclei with figures of mitosis. The cytoplasm was abundant and eosinophilic. (C): The cancer cells were positive for P40 (immunohistochemistry (IHC), 20 ×). (D): The cancer cells were positive for CK5/6 (IHC, 20 ×); (E): The cancer cells were negative for CDX2 with positive internal control of colonic mucosal epithelial cells (IHC), 20 ×).

Histopathology sections (Figures 2A, 2B) showed a colonic mucosa largely infiltrated by a carcinomatous tumoral proliferation consisting of sheets and nests of large polygonal tumor cells, showing hyperchromatic, anisocaryotic nuclei with figures of mitosis. The cytoplasm was abundant and eosinophilic. The surrounding stroma was fibrotic, with a sparse inflammatory infiltrate with variable keratinization (keratin pearls) produced by tumor cells. Immunohistochemically, the tumor cells were positive for the squamous markers CK5/6 and p40 (Figures 2C, 2D). They were negative for CDX2 (Figure 2E).

The histopathology report of the patient suggested a sigmoid localization of moderately differentiated keratinizing squamous cell carcinoma. Considering the rarity of primary SCC of the colon, the pathology report indicates that the primary nature of the tumor should only be retained after excluding the possibility of SCC metastasis or direct invasion of other organs.

The patient underwent an extensive evaluation to identify a possible SCC metastasis from other organs like the oropharynx, larynx, esophagus, lungs, anal canal, and skin. Their evaluations (chest, abdominal, and pelvic CT scans) were negative. The diagnosis of primary sigmoid SCC was established.

After a multidisciplinary discussion, the patient underwent a resection of the sigmoid with lymph node dissection. Subsequently, functional end‑to‑end anastomosis was performed. The patient had an uneventful postoperative recovery. The histopathological examination of the resected specimen was in accordance with the diagnosis of moderately differentiated SCC of the sigmoid.

The tumor invades through the muscularis propria into the peri-colorectal tissues. Perineural invasion was present without lymphovascular invasion. Proximal and distal resection margins were free of tumors. There were no metastatic lymph nodes (twelve lymph nodes were examined). The cancer was classified, according to the eighth edition of tumor nodes. metastasis (TNM) classification, as pT3 N0 M0 (Stage IIA).

After a multidisciplinary discussion, the oncologists decide on an active follow-up of the patient without any further treatment.

## Discussion

Colorectal cancer (CRC) is the third most common cancer in the world [4]. In 2020, there will be over 1.93 million new cases of colorectal cancer and 0.94 million CRC-caused deaths [4]. More than 90% of CRCs are adenocarcinomas that develop from epithelial cells of the colorectal mucosa [5]. Other uncommon colorectal cancer histological types include neuroendocrine, adenosquamous, spindle cell, and undifferentiated carcinomas [6]. On the other hand, primary squamous cell carcinoma (SCC) of the colon is an uncommon histological type. The first colonic SCC case was published in the medical literature by Schmidtian in 1919 [7]. Raiford reported the first rectal SCC in 1933 [8]. 

To our knowledge, about 150 cases have been reported in the literature [9]. It occurs at a rate of 0.10 to 0.25 per 1,000 colorectal cancers [10]. Dimitrios et al. published the largest systematic review of the literature concerning the SCC of the colon and rectum. They implicated 99 patients, 45 (45.5%) males, and 54 (54.5%) females, with an average age of 56.98 ± 12.19 years (mean, SD) [3].

A review of the literature revealed that tumor can occur in any part of the colorectum and affects mostly the recto-sigmoid part of the colon followed by the right hemicolon and SCC in the left hemicolon is very rare [11].

Clinically, colorectal SCC symptoms are practically identical to those of colorectal adenocarcinoma, including gastrointestinal bleeding, abdominal pain, weight loss, anorexia, constipation, and diarrhea [12]. However, colorectal SCC can be discovered with some complications such as acute abdominal and intestinal obstruction in our case.

The pathophysiology of colorectal SCC is still unknown. A number of hypotheses have been proposed. The presence of pluripotent stem cell differentiation in the colonic mucosa competent multidirectional may cause the formation of squamous epithelium capable of malignant transformation [13]. Another hypothesis is that squamous metaplasia of the glandular epithelium is caused by numerous pathological situations such as chronic inflammation due to radiation therapy, inflammatory bowel disease, and ulcerative colitis. Parasitic and viral infections have been implicated in the carcinogenesis of colorectal SCC such as Schistosomiasis, Entamoeba histolytica, and human papillomavirus (HPV), but the association is not fully demonstrated. [14,15]. In this case, the patient had no specific past medical history and no evidence of any predisposing factor previously described.

Colorectal carcinomas are diagnosed and confirmed by histopathological examination, which is usually performed on endoscopic biopsy samples rather than surgical parts. In our case, the diagnosis was established on the sigmoidectomy specimen.

The diagnosis of primary colorectal SCC should not be established only after satisfaction of the following criteria: First, the presence of pathological features and immunohistochemical profile of SCC without glandular differentiation. Second, excluding the possibility of SCC metastasis or direct invasion of other tissues or organs. Third, there is no squamous-lined fistula tract to the affected bowel. Fourth, absence of tumor extension from the anal squamous epithelium [16].

The pathological features of the primary colorectal SCC are similar to those seen in other organs such as the skin, esophagus, anus, lung, and cervix. Morphologically, the tumor is generally organized into nests and lobules composed of atypical polygonal tumor cells and is graded as well-differentiated, moderately differentiated, or poorly differentiated carcinoma [17]. In some cases, particularly if the tumor is poorly or undifferentiated, an immunohistochemical study may be required to confirm the diagnosis, the tumor cells must express squamous differentiation antibodies including P40, P63, and cytokeratin 5/6 (As in our case).

Colorectal adenocarcinoma has benefited from a remarkable revolution in the therapeutic arsenal in recent decades, with multiple treatment options and well-defined and standardized therapeutic guidelines including surgery, chemotherapy, immunotherapy, targeted therapy, and radiotherapy [18]. However, in light of its rarity, the standard treatment for colorectal SCC has yet to be determined. The majority of authors agree that surgery is the main treatment for primary SCC of the colon [19]. Given the tumor's rarity, the efficacy and duration of adjuvant chemotherapy or radiation have not been established. Gelas et al. report that the ideal treatment for CTS of the colon is based on neoadjuvant chemoradiation followed by surgical resection of the tumor [2]. Several therapeutic protocols for colorectal SCC are described in the literature but apparently without influence on the outcome [3].

Colorectal SCC is presumed to be more aggressive and to have a worse prognosis than colorectal adenocarcinoma [20]. Due to the tumor's rarity, prognostic factors and exact overall survival for each stage of the disease are yet unknown.

## Conclusions

Primary colorectal squamous cell carcinoma is an extremely rare histological type with unclear pathogenesis. Our knowledge of this entity is still obscure. There's no current agreement on a specific management approach for these cases. More research and large case series are needed to better understand this tumor and establish standardized therapeutic management.

## References

[1] Gordon PH, Nivatvongs S (2007). Principles and Practice of Surgery for the Colon, Rectum, and Anus. Principles and Practice of Surgery for the Colon, Rectum, and Anus (3rd.

[2] Gelas T, Peyrat P, Francois Y (2002). Primary squamous-cell carcinoma of the rectum: report of six cases and review of the literature. Dis Colon Rectum.

[3] Schizas D, Katsaros I, Mastoraki A (2022). Primary Squamous Cell Carcinoma of Colon and Rectum: A Systematic Review of the Literature. J Invest Surg.

[4] Xi Y, Xu P (2021). Global colorectal cancer burden in 2020 and projections to 2040. Transl Oncol.

[5] Ahadi M, Sokolova A, Brown I, Chou A, Gill AJ (2021). The 2019 World Health Organization classification of appendiceal, colorectal and anal canal tumours: an update and critical assessment. Pathology.

[6] Fleming M, Ravula S, Tatishchev SF, Wang HL (2012). Colorectal carcinoma: pathologic aspects. J Gastrointest Oncol.

[7] Schmidtmann M (1919). Zur Kenntnis seltener Krebsformen (Article in German). Virchows Arch Pathol Anat Physiol Klin Med.

[8] Lannaz S, Elomrani F, ouziane I, Mrabti H and Errihani H (2015). Squamous cell carcinoma of the colon: a case report and literature review. Austin J Clin Med.

[9] G Jahromi N (2020). Primary squamous cell carcinoma of the descending colon. Cureus.

[10] Dyson T, Draganov PV (2009). Squamous cell cancer of the rectum. World J Gastroenterol.

[11] Li XY, Teng G, Zhao X, Zhu CM (2022). Primary sigmoid squamous cell carcinoma with liver metastasis: a case report. World J Clin Cases.

[12] Bouzroud M, Azzakhmam M, El Mehdi A, Mohammed BS, Hakim EK, Mohaned O, Ahmed B (2021). Primary cecal squamous cell carcinoma: a case report of a rare tumor with poor prognosis. Case Rep Oncol Med.

[13] Palvio DH, Sørensen FB, Kløve-Mogensen M (1985). Stem cell carcinoma of the colon and rectum. Report of two cases and review of the literature. Dis Colon Rectum.

[14] Vezeridis MP, Herrera LO, Lopez GE, Ledesma EJ, Mittleman A (1983). Squamous-cell carcinoma of the colon and rectum. Dis Colon Rectum.

[15] Sotlar K, Köveker G, Aepinus C, Selinka HC, Kandolf R, Bültmann B (2001). Human papillomavirus type 16-associated primary squamous cell carcinoma of the rectum. Gastroenterology.

[16] Williams GT, Blackshaw AJ, Morson BC (1979). Squamous carcinoma of the colorectum and its genesis. J Pathol.

[17] Que SK, Zwald FO, Schmults CD (2018). Cutaneous squamous cell carcinoma: Incidence, risk factors, diagnosis, and staging. J Am Acad Dermatol.

[18] Argilés G, Tabernero J, Labianca R (2020). Localised colon cancer: ESMO Clinical Practice Guidelines for diagnosis, treatment and follow-up. Ann Oncol.

[19] Dikshit V, Ali I, Patil C, Manerikar K, Mody P (2019). Squamous cell carcinoma of colon-an etiopathological surprise. J Gastrointest Cancer.

[20] Comer TP, Beahrs OH, Dockerty MB (1971). Primary squamous cell carcinoma and adenocanthoma of the colon. Cancer.

